# 3D-AttenNet model can predict clinically significant prostate cancer in PI-RADS category 3 patients: a retrospective multicenter study

**DOI:** 10.1186/s13244-024-01896-1

**Published:** 2025-01-29

**Authors:** Jie Bao, Litao Zhao, Xiaomeng Qiao, Zhenkai Li, Yanting Ji, Yueting Su, Libiao Ji, Junkang Shen, Jiangang Liu, Jie Tian, Ximing Wang, Hailin Shen, Chunhong Hu

**Affiliations:** 1 Department of Radiology, The First Affiliated Hospital of Soochow University, Suzhou, China; 2 School of Engineering Medicine, Beihang University, Beijing, China; 3 Key Laboratory of Big Data-Based Precision Medicine (Beihang University), Ministry of Industry and Information Technology of China, Beijing, China; 4 Department of Radiology, Suzhou Kowloon Hospital, Shanghai Jiaotong University School of Medicine, Suzhou, China; 5 Department of Radiology, The Affiliated Zhangjiagang Hospital of Soochow University, Zhangjiagang, China; 6 Department of Radiology, The People’s Hospital of Taizhou, Taizhou, China; 7 Department of Radiology, Changshu No.1 People’s Hospital, Changshu, China; 8 Department of Radiology, The Second Affiliated Hospital of Soochow University, Suzhou, China

**Keywords:** Deep learning, MRI, Clinically significant prostate cancer, PI-RADS

## Abstract

**Purposes:**

The presence of clinically significant prostate cancer (csPCa) is equivocal for patients with prostate imaging reporting and data system (PI-RADS) category 3. We aim to develop deep learning models for re-stratify risks in PI-RADS category 3 patients.

**Methods:**

This retrospective study included a bi-parametric MRI of 1567 consecutive male patients from six centers (Centers 1–6) between Jan 2015 and Dec 2020. Deep learning models with double channel attention modules based on MRI (AttenNet) for predicting PCa and csPCa were constructed separately. Each model was first pretrained using 1144 PI-RADS 1–2 and 4–5 images and then retrained using 238 PI-RADS 3 images from three training centers (centers 1–3), and tested using 185 PI-RADS 3 images from the other three testing centers (centers 4–6).

**Results:**

Our AttenNet models achieved excellent prediction performances in testing cohort of center 4–6 with the area under the receiver operating characteristic curves (AUC) of 0.795 (95% CI: [0.700, 0.891]), 0.963 (95% CI: [0.915, 1]) and 0.922 (95% CI: [0.810, 1]) in predicting PCa, and the corresponding AUCs were 0.827 (95% CI: [0.703, 0.952]) and 0.926 (95% CI: [0.846, 1]) in predicting csPCa in testing cohort of center 4 and center 5. In particular, 71.1% to 92.2% of non-csPCa patients were identified by our model in three testing cohorts, who can spare from invasive biopsy or RP procedure.

**Conclusions:**

Our model offers a noninvasive screening clinical tool to re-stratify risks in PI-RADS 3 patients, thereby reducing unnecessary invasive biopsies and improving the effectiveness of biopsies.

**Critical relevance statement:**

The deep learning model with MRI can help to screen out csPCa in PI-RADS category 3.

**Key Points:**

- AttenNet models included channel attention and soft attention modules.
- 71.1–92.2% of non-csPCa patients were identified by the AttenNet model.
- The AttenNet models can be a screen clinical tool to re-stratify risks in PI-RADS 3 patients.

**Graphical Abstract:**

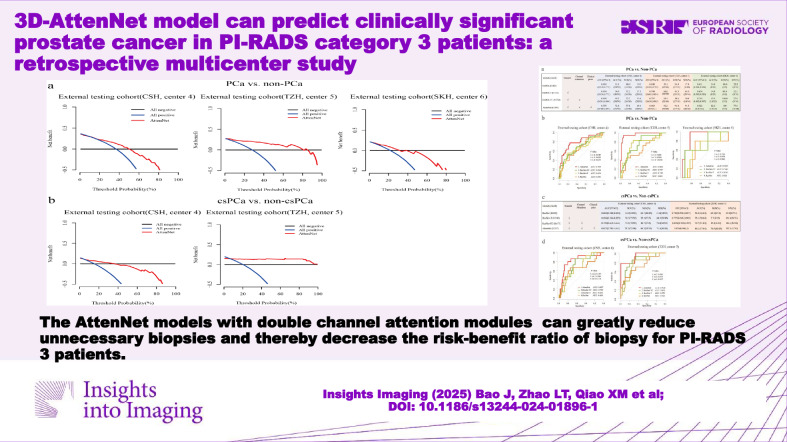

## Background

The diagnosis and management of prostate cancer (PCa) have substantially evolved over the last few decades [1]. At present, transrectal ultrasound-guided biopsy, fusion biopsy, and prostate-specific antigen (PSA) are the most common tools for earlier diagnosis or screening of PCa. Yet, these methods often yield false positive results or missing diagnoses, while the biopsy is somewhat invasive and not always well accepted by patients [2].

Multiparametric magnetic resonance imaging (mpMRI) of the prostate has been widely utilized for the detection and risk stratification of clinically significant prostate cancer (csPCa) [3–5]. According to the prostate imaging reporting and data system version 2.1 (PI-RADS v2.1) [6], prostatic lesions detected on mpMRI are scored 1–5. PI-RADS 1–2 indicates very low and low probabilities of having csPCa, whereas PI-RADS 4–5 suggests a high probability [6, 7]. As for lesions with PI-RADS 3, the presence of csPCa is considered to be equivocal [6]. In clinical practice, PI-RADS 3 patients usually require biopsies. However, some recent studies suggest that most PI-RADS 3 lesions do not contain csPCa [8], which poses the question of whether or not a biopsy is really necessary [9].

To overcome the shortcomings of the semi-quantification of PI-RADS assessment, recent studies have used radiomics methods to discriminate between csPCa and non-csPCa for PI-RADS 3 patients [10–14]. However, extracting radiomics features greatly relies on the slice-by-slice manual delineation of lesions in MR images, which is time-consuming and laborious, particularly when a larger number of samples are used to construct radiomics models [15, 16].

Deep learning (DL) can mine information in images and characterize the intrinsic features of tumors, accurately reflecting tumors’ spatial heterogeneity [17, 18]. Compared to the radiomics methodology, DL methods are almost independent of the manual delineation of tumors and can provide automatic analysis of medical images and have also been widely used in recent years to detect PCa [19–22]. These studies use DL methods to detect csPCa lesions regardless of the PI-RADS scores [6, 7], but none was specifically focused on differentiating amongst PI-RADS 3 lesions. Indeed, whether a DL model could provide incremental value to precisely screen out csPCa from those equivocal lesions is yet unknown. Therefore, it is very likely that the decisive features for detecting csPCa from these patients are much subtler than those with the other PI-RADS categories (i.e., PI-RADS 1, 2, 4, and 5). Thus, developing a new DL model sensitive to these subtle decisive features is necessary to detect csPCa in PI-RADS 3 patients.

Indeed, this study used datasets from multiple centers to construct novel DL models with double attention modules for predicting csPCa and PCa in equivocal PI-RADS 3 patients.

## Materials and methods

### Patients

**Fig. 1:**
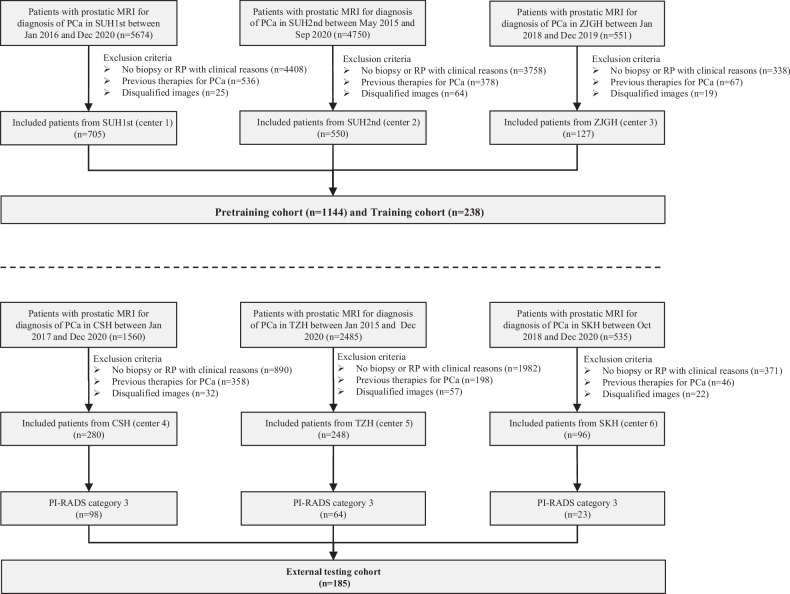
Flowchart of the inclusion criteria and exclusion criteria of the multicenter study. center 1, SUH1st, the first affiliated hospital of Soochow University; center 2, SUH2nd, the second affiliated hospital of Soochow University; center 3, ZJGH, People’s Hospital of Zhangjiagang; center 4, CSH, Changshu no. 1 People’s Hospital; center 5, TZH, People’s Hospital of Taizhou; center 6, SKH, Suzhou Kowloon Hospital; PCa, prostate cancer

Patients suspected of PCa from six centers were enrolled between Jan 2015 and Dec 2020. The inclusion criteria were the following: (1) those who underwent standard prostate 3.0-T MRI within 4 weeks before biopsy; (2) those who underwent standard transrectal ultrasonography (TRUS)/MRI fusion or cognitive fusion targeted biopsy and systematic biopsy. Exclusion criteria were: (1) Incomplete MRI sequence or poor image quality (displacement or motion artifacts); (2) previous history of biopsy or surgery or treatment for PCa. Table S1 summarizes the baseline characteristics of the patients from the six centers. The study flowchart is shown in Fig. 1.

The Ethics Committee of center 1 approved this study, and the requirement of written informed consent was waived.

### Prostatic MRI and PI-RADS assessment

All examinations were performed on 3.0-T MRI scanners with pelvic phased-array coils at six centers. The details of the MRI parameters are shown in Table S2 and Supplementary Section 1.

The index lesions in multiparametric prostate MRI images of all included patients were assessed by five board-certified radiologists and two expert-level radiologists according to the PI-RADS v2.1 [6]. The details of the PI-RADS assessment are described in Supplementary Section 2. Finally, of the 2006 patients, 78.9% (1583/2006) were patients with PI-RADS 1, 2, 4, and 5, and 21.1% (423/2006) were patients with PI-RADS 3. Table 1 summarizes the baseline characteristics of PI-RADS category 3 patients for each center.

**Table 1:** The baseline characteristics of the PI-RADS category 3 patients in training cohorts and external testing cohorts

Variable	Training cohorts (centers 1–3, *n* = 238)				External testing cohorts (centers 4–6, *n* = 185)				
	SUH1st (center 1, *n* = 82)	SUH2nd (center 2, *n* = 129)	ZJGH (center 3, *n* = 27)	Total	CSH (center 4, *n* = 98)	TZH (center 5, *n* = 64)	SKH (center 6, *n* = 23)	Total	*p*
Number of subjects	82	129	27	238	98	64	23	185	
Age (y), mean	68.5 ± 3.5	70.0 ± 7.8	78.0 ± 7.0	70.0 ± 7.8	69.9 ± 6.7	71.1 ± 8.2	70.3 ± 6.9	70.4 ± 7.3	0.231
PSA level, median, (IQR)	10.5 (6.7–16.1)	11.1 (6.9–15.7)	11.2 (9.4–24.8)	11.1 (7.1–14.5)	11.4 (7.1–18.2)	8.2 (5.3–16.6)	10.1 (6.3–14.8)	9.9 (6.3–18.0)	0.445
D-max (mm), median (IQR)	19.8 (16.5–26.8)	18.3 (13.8–23.2)	19.6 (11.9–28.2)	18.8 (14.5–24.9)	15.7 (12.5–20.7)	35.7 (21.0–45.4)	10.0 (7.5–12.5)	17.2 (12.8–31.0)	0.467
Prostate zone, *n*	82	129	27	238	98	64	23	185	0.918
PZ, *n* (%)	20 (24.4%)	24 (18.6%)	8 (29.6%)	52 (21.8%)	28 (28.6%)	3 (4.7%)	3 (13.0%)	34 (18.4%)	
TZ, *n* (%)	59 (72.0%)	82 (63.6%)	19 (44.4%)	160 (67.2%)	68 (69.4%)	49 (76.6%)	20 (87.0%)	137 (74.1%)	
PZ and TZ, *n* (%)	3 (3.6%)	23 (17.8%)	0 (0.0%)	26 (10.9%)	2 (2.0%)	12 (18.8%)	0 (0.0%)	14 (7.6%)	
Biopsy ISUP grade, *n* (%)	81	129	27	237	96	64	22	182	0.929
GG0 (benign)	44 (54.3%)	100 (77.5%)	10 (37.0%)	154 (65.0%)	62 (64.6%)	46 (71.9%)	17 (77.3%)	125 (68.7%)	
GG1	8 (9.9%)	23 (17.8%)	11 (40.7%)	42 (17.7%)	4 (4.2%)	1 (1.6%)	3 (13.6%)	8 (4.4%)	
GG2	12 (14.8%)	3 (2.3%)	4 (14.8%)	19 (8.0%)	16 (16.7%)	4 (6.3%)	2 (9.1%)	22 (12.1%)	
GG3	11 (13.6%)	2 (1.6%)	0 (0.0%)	13 (5.5%)	8 (8.3%)	7 (10.9%)	0 (0.0%)	15 (8.2%)	
GG4	4 (4.9%)	0 (0.0%)	2 (7.4%)	6 (2.5%)	5 (5.2%)	3 (4.7%)	0 (0.0%)	8 (4.4%)	
GG5	2 (2.5%)	1 (0.8)	0 (0.0%)	3 (1.3%)	1 (1.0%)	3 (4.7%)	0 (0.0%)	4 (2.2%)	
Surgical ISUP grade, *n* (%)	33	18	8	59	2	3	3	8	< 0.001
GG1	5 (15.2%)	14 (77.8%)	5 (62.5%)	24 (40.7%)	0 (0.0%)	0 (0.0%)	1 (33.3%)	1 (12.5%)	
GG2	12 (36.4%)	2 (11.1%)	2 (25.0%)	16 (27.1%)	1 (50.0%)	2 (66.7%)	2 (66.7%)	5 (62.5%)	
GG3	9 (27.3%)	2 (11.1%)	1 (12.5%)	12 (20.3%)	1 (50.0%)	1 (33.3%)	0 (0.0%)	2 (25.0%)	
GG4	0 (0.0%)	0 (0.0%)	0 (0.0%)	0 (0.0%)	0 (0.0%)	0 (0.0%)	0 (0.0%)	0 (0.0%)	
GG5	7 (21.2%)	0 (0.0%)	0 (0.0%)	7 (11.9%)	0 (0.0%)	0 (0.0%)	0 (0.0%)	0 (0.0%)	
Label, *n* (%)	82	129	27	238	98	64	23	185	0.251
non-PCa	44 (53.7%)	100 (77.5%)	10 (37.0%)	154 (64.7%)	62 (63.3%)	46 (71.9%)	18 (78.3%)	126 (68.1%)	
PCa	38 (46.3%)	29 (22.5%)	17 (63.0%)	84 (35.3%)	36 (36.7%)	18 (28.1%)	5 (21.7%)	59 (31.9%)	
Non-csPCa	64 (78.0%)	123 (95.3%)	24 (88.9%)	211 (88.7%)	83 (84.7%)	51 (79.7%)	23 (100%)	157 (84.9%)	
csPCa	18 (22.0%)	6 (4.7%)	3 (11.1%)	27 (11.3%)	15 (15.3%)	13 (20.3%)	0 (0.0%)	28 (15.1%)	
ECE, *n* (%)	33	18	8	59	2	3	3	8	0.837
Present	5 (15.2%)	4 (22.2%)	0 (0.0%)	9 (15.3%)	0 (100%)	1 (33.3%)	0 (0.0%)	1 (12.5%)	
Absent	28 (84.8%)	14 (77.8%)	8 (100%)	50 (84.7%)	2 (0.0%)	2 (66.7%)	3 (100.0%)	7 (87.5%)	
SVI, *n* (%)	33	18	8	59	2	3	3	8	0.514
Present	3 (9.1%)	0 (0.0%)	0 (0.0%)	3 (5.1%)	0 (0.0%)	0 (0.0%)	0 (100%)	0 (0.0%)	
Absent	30 (90.9%)	18 (100%)	8 (100%)	56 (94.9%)	2 (100%)	3 (100%)	3 (100.0%)	8 (100.0%)	
LNI, *n* (%)	15	1	1	17	1	3	1	5	1.000
Present	0 (0.0%)	0 (0.0%)	0 (0.0%)	0 (0.0%)	0 (0.0%)	0 (0.0%)	0 (0.0%)	0 (0.0%)	
Absent	15 (100.0%)	1 (100%)	1 (100%)	17 (100.0%)	1 (100%)	3 (100%)	1 (100%)	5 (100%)	

Unless indicated otherwise, data are numbers of patients with percentages in parentheses. The *p*-value was evaluated by a two-tailed *t*-test with unequal variance. Gleason grade (GG) is according to the 2014 International Society of Urological Pathology (ISUP) standards

*PSA* prostate-specific antigen, *ciPCa* clinically insignificant prostate cancer, *csPCa* clinically significant prostate cancer, *PZ* peripheral zone, *TZ* transition zone, *CZ* center zone, *AFMS* anterior fibromuscular stroma, *ECE* extracapsular extension, *SVI* seminal vesicle infiltration, *LNI* lymph node invasion, *D-max* diameter in greatest dimension; *center 1, SUH1st*, the first affiliated hospital of Soochow Universityxxx; *center 2, SUH2nd*, the second affiliated hospital of Soochow University; *center 3, ZJGH*, People’s Hospital of Zhangjiagang; *center 4, CSH*, Changshu No.1 People’s Hospital; *center 5, TZH*, People’s Hospital of Taizhou; *center 5, CSH*, Changshu No.1 People’s Hospital; *center 6, SKH*, Suzhou Kowloon Hospital

## Histopathology

Patients with PI-RADS category ≥ 3 underwent targeted MRI-guided biopsy in conjunction with systemic transrectal ultrasound (TRUS)-guided biopsy or cognitive fusion biopsy in conjunction with systemic TRUS-guided biopsy; patients with PI-RADS 1–2 underwent only systemic TRUS-guided biopsy. In terms of histopathology, patients with ISUP ≥ 3 were defined as having csPCa, and those with ISUP ≥ 1 were defined as having PCa [8, 23]. The details of the histological review are described in Supplementary Section 3. Table 1 summarizes the details of the results of the histopathology assessment for each of the 6 centers.

### Study cohort

Patients with PI-RADS 1, 2, 4, and 5 of centers 1–3 were used as a pretraining cohort (total *N* = 1144), and PI-RADS 3 patients from the same centers were used as the training cohort (total *N* = 238). For centers 4–6, PI-RADS 3 patients were used as external testing cohorts (total *N* = 185) (Fig. 1), and PI-RADS 1, 2, 4, and 5 patients (*N* = 439) were not applied in the present study. In each pretraining and training cohort, the patients were randomly divided into two datasets, including nine-tenth (i.e., the training dataset) and one-tenth patients (i.e., the tuning dataset), which were used to train the network weights and optimize the hyperparameters of the DL model, respectively.

### Prostate MRI data preprocessing

The preprocessing of prostate MRI images included data de-identification, registration, data harmonization, and data augmentation (Supplementary Section 4) referring to our previous study [22].

### AttenNet model development

#### Model architecture

**Fig. 2:**
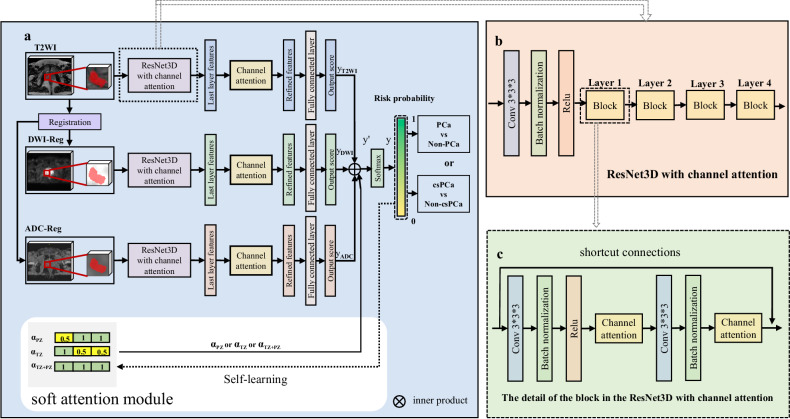
Flowchart of the development of the AttenNet models. **a** The architecture of the AttenNet Model with channel attention and soft attention module. First, the VOIs were fed into a module of ResNet3D with channel attention; then, the three parallel and independent branches were used as inputs; finally, the three branches were integrated into a fusion network with a soft attention module. **b** Details of ResNet3D with channel attention. **c** Details of the block in the ResNet3D with channel attention. PCa, prostate cancer; csPCa, clinically significant prostate cancer; PZ, peripheral zone; TZ, transitional zone; Self-learning, the automatic and iterative change in the values of the elements of embedding vectors during the pretraining and training processes

In the present study, two DL models were developed separately to identify PCa and csPCa. These two models had the same architectures, which were referred to as AttenNet because they both included the channel attention and soft attention modules. As shown in Fig. 2, the AttenNet model consisted of three parallel and independent branches with the 3D regions of interest of T2WI, DWI images, and ADC maps as inputs, respectively, which used the state-of-art ResNet3D as the basic network due to its ability to mine features of deep layers and generate accurate predicting values using shortcut connections [24].

A fusion network integrated these three branches with the involvement of the soft attention module (Fig. 2a). Recent studies [25] have suggested a difference in the characteristics of cancer between the peripheral zone (PZ) and transitional zone (TZ) of the prostate. Further, according to PI-RADS V2.1 [6], the PI-RADS assessment category of lesions in TZ and PZ are dominantly dependent on T2WI and DWI scores, respectively. Thus, in the present study, we used the soft attention module to provide the location information of prostate lesions for the AttenNet model. This information was used as a clinical prior and can highlight the role of the corresponding modality of images (i.e., T2WI, or/and DWI and ADC) for the classification between csPCa and non-csPCa or that between PCa and non-PCa (More details were in Supplementary Materials Section 5).

#### Training strategy

The present study employed a transfer learning strategy to develop DL models. Specifically, the DL model was first trained using a pretraining cohort (i.e., the patients with PI-RADS 1, 2, 4, and 5). Then DL model was retrained using training cohorts and tested using testing cohorts, which included only PI-RADS 3 patients. In this way, the knowledge learned from the pretraining cohort was transferred to the retraining step using the training cohort of PI-RADS 3. Thus, using the transfer learning strategy, patients with PI-RADS 3 and those with PI-RADS 1,2, 4, and 5 were contributed to develop the DL model for the detection of csPCa or PCa in PI-RADS 3 patients. Such a transfer learning strategy may improve the performance of the DL model.

### Statistical analysis

Independent *t*-tests were used to compare normally distributed continuous variables. Mann–Whitney *U*-tests were used to compare non-normally distributed continuous variables. Chi-squared test was applied to assess the categorical variables. Quantitative variables were expressed as mean ± standard deviation (mean ± SD) or median and interquartile range (IQR) as appropriate.

Receiver operating characteristic (ROC) curves were depicted to evaluate the classification performance of the proposed models. In each external testing cohort, the ROC curves of the models were compared using Delong tests, and sensitivity (SEN), specificity (SPE), and accuracy (ACC) were calculated at a threshold that maximized the value of the Youden index in the tuning dataset of training cohort. Decision curve analysis (DCA) was used to evaluate the net benefit of the patients using the biopsy strategy based on the proposed model in the present study. The statistical analysis was conducted with Python (https://www.python.org, version 3.8.3) and MedCalc software (Ostend, Belgium, https://www.medcalc.org, version 19.6.4). A two-sided *p* < 0.05 was considered statistically significant in all statistical tests.

## Results

### Baseline characteristics

In the training cohort (centers 1–3), 35.3% (84/238) and 64.7% (154/238) patients were diagnosed as PCa and benign (non-PCa), respectively and 11.3% (27/238) and 88.7% (211/238) as csPCa and non-csPCa, respectively. The positive/negative number and ratio concerning PCa and csPCa for each training and testing cohort are shown in Table 1. More details are in Supplementary Materials Section 6.

### Diagnostic performance of DL models for predicting PCa and csPCa in PI-RADS 3 patients

**Fig. 3:**
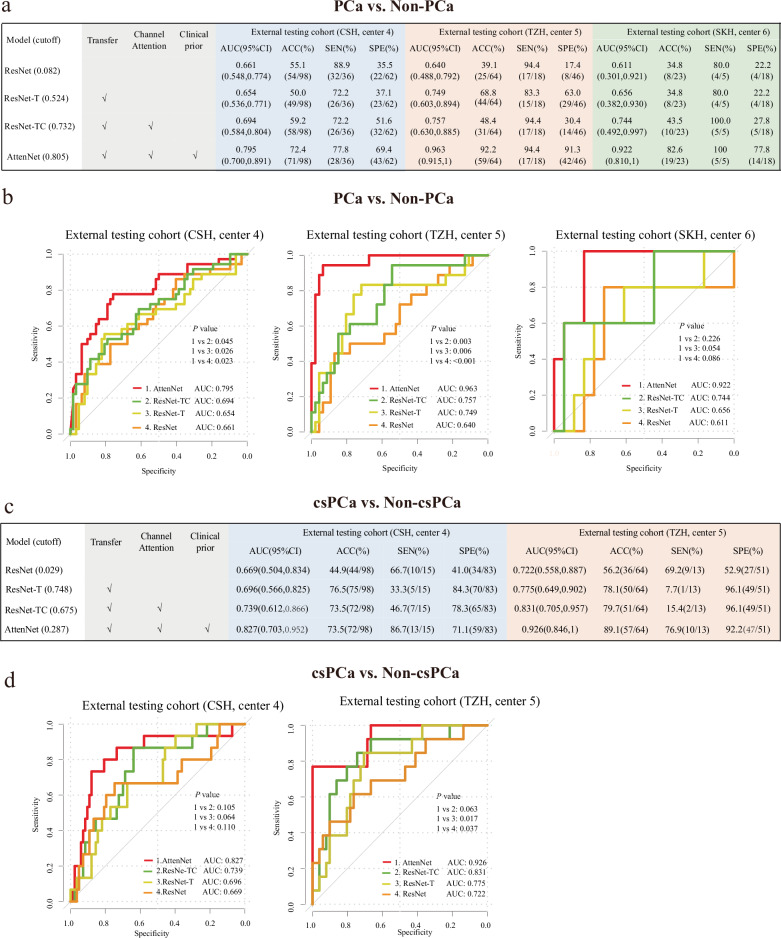
Diagnosis performances of different deep learning models for predicting PCa and csPCa. **a** Diagnosis performances of the ResNet, ResNet-T, ResNet-TC, and AttenNet models for predicting PCa in three external testing cohorts. The AttenNet model yields the highest AUC compared with the other three models in predicting PCa. **b** ROC curves of the AttenNet model and the other three models for predicting PCa in three external testing cohorts. **c** Diagnosis performances of the ResNet, ResNet-T, ResNet-TC, and AttenNet models for predicting csPCa in two external testing cohorts. Similarly, the AttenNet model yields the highest AUC among all the models in predicting csPCa. **d** ROC curves of the AttenNet model and the other three models for predicting csPCa in two external testing cohorts. ROC, receiver operating characteristics; AUC, area under ROC curve; ACC, accuracy; SEN, sensitivity; SPE, specificity; center 4, TZH, People’s Hospital of Taizhou; center 5, CSH, Changshu No.1 People’s Hospital; center 6, SKH, Suzhou Kowloon Hospital; PCa, prostate cancer; csPCa, clinically significant prostate cancer; ResNet-T, ResNet with transfer module; ResNet-TC, ResNet with transfer and channel attention modules; AttenNet, ResNet combined with transfer, channel attention and soft attention modules

To evaluate the performance of the proposed AttenNet models in predicting PCa and csPCa in PI-RADS 3 patients, an ablation experiment of the models with different modules was performed. As shown in Fig. 3, we compared the performance of models among the ResNet, ResNet combined with transfer module (ResNet-T), ResNet combined with transfer and channel attention modules (ResNet-TC), and AttentNet that referred to the ResNet combined with a transfer, channel attention, and soft attention modules. For the prediction of PCa, the AttenNet model achieved an area under the ROC curve (AUC) of 0.795 (95% CI: [0.700, 0.891]), 0.963 (95% CI: [0.915,1.00]), and 0.922 (95% CI: [0.810, 1.00]) in the external testing cohorts of center 4, center 5, and center 6, respectively (Fig. 3a). Furthermore, the AUC of the AttenNet model was significantly higher than those of the other models (i.e., ResNet, ResNet-T, and ResNet-TC) in center 4 (AUC = 0.661, 0.654, and 0.694, respectively) and center 5 (AUC = 0.640, 0.749, and 0.757, respectively) (*P*s < 0.05, Fig. 3b). Although no difference in AUC was observed between the AttenNet (AUC = 0.922) and ResNet, ResNet-T, and ResNet-TC in center 6 (AUC = 0.611, 0.656, and 0.744, respectively) (*P*s > 0.05, Fig. 3b), the AUC of the former was numerically higher than those of the latter. Thus, in terms of AUC, the AttenNet model showed the best performance for predicting PCa among these four DL models. Furthermore, in the external testing cohorts of center 4, center 5, and center 6, the AttenNet model achieved an ACC of 72.4% (71/98), 92.2% (59/64), and 82.6% (19/23); an SEN of 77.8% (28/36), 94.4% (17/18), and 100% (5/5); SPE of 69.4% (43/62), 91.3% (42/46), and 77.8% (14/18), respectively (Fig. 3a).

As shown in Fig. 3c, for the prediction of csPCa, the AttenNet model achieved an AUC of 0.827 (95% CI: [0.703, 0.952]) and 0.926 (95% CI: [0.846, 1]) in the external testing cohorts of center 4 and center 5, respectively. As confirmed by the pathological exam, all PI-RADS 3 patients in the external testing cohort of center 6 were non-csPCa patients, and therefore the AUC for predicting csPCa in this cohort could not be calculated due to the lack of binary labels. Thus, the patients of this cohort were not used to test the prediction of csPCa. Further, in center 5, the AUC of the AttenNet model (AUC = 0.926) was significantly higher than those of the ResNet (AUC = 0.722, *p* = 0.037) and ResNet-T (AUC =0.775, *p* = 0.017), and marginally higher than that of ResNet-TC (AUC = 0.831, *p* = 0.063). Although no significant difference in AUC was observed between the AttenNet model (AUC = 0.827) and each of ResNet, ResNet-T, and ResNet-TC in center 4 (AUC = 0.669, 0.696, and 0.739, respectively) (*P*s > 0.05, Fig. 3d), the AUC of the former was numerically higher than those of the latter (Fig. 3d). Thus, in terms of AUC, the AttenNet model showed the best performance for predicting csPCa among these four DL models. In addition, in the external testing cohorts of center 4 and center 5, the AttenNet model achieved an ACC of 73.5% (72/98) and 89.1% (57/64); a SEN of 86.7% (13/15) and 76.9% (10/13); a SPE of 71.1% (59/83) and 92.2% (47/51), respectively (Fig. 3c).

**Fig. 4:**
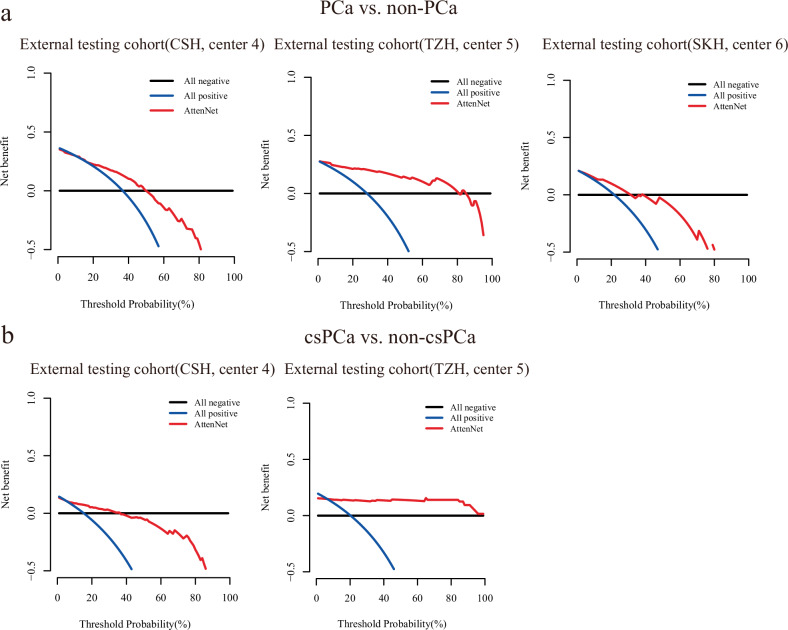
Results of DCA of AttenNet for predicting PCa and csPCa. **a** Results of DCA of AttenNet for predicting PCa in three external testing cohorts. **b** Results of DCA of AttenNet for predicting csPCa in two external testing cohorts. The red lines indicate the net benefit of patients when using a biopsy based on the AttenNet model for detecting PCa (**a**) and csPCa (**b**). The blue lines indicate the net benefit of patients when they were all predicted to be positive (i.e., all patients with PI-RADS 3 underwent the biopsy) for the detection of PCa (**a**) and csPCa (**b**). The black lines indicate the net benefit of patients when they were all predicted to be negative for the detection of PCa (**a**) and csPCa (**b**)

As revealed by DCA, the biopsy strategy based on the AttenNet model shows greater net benefit than that based on PI-RADS assessment (i.e., all PI-RADS 3 patients underwent the biopsy) for both detections of PCa (Fig. 4a) and csPCa (Fig. 4b) in the external testing cohorts. As shown in Fig. 4, the biopsy strategy based on the AttenNet model shows greater net benefit (Red line in Fig. 4) than that based on PI-RADS assessment (i.e., all patients with PI-RADS category 3 underwent the biopsy in the present study) (Blue line in Fig. 4) for detections of PCa (Fig. 4a) and csPCa (Fig. 4b) in each external cohort.

### Clinical practice of the AttenNet models for predicting PCa and csPCa in PI-RADS category 3 patients

**Fig. 5:**
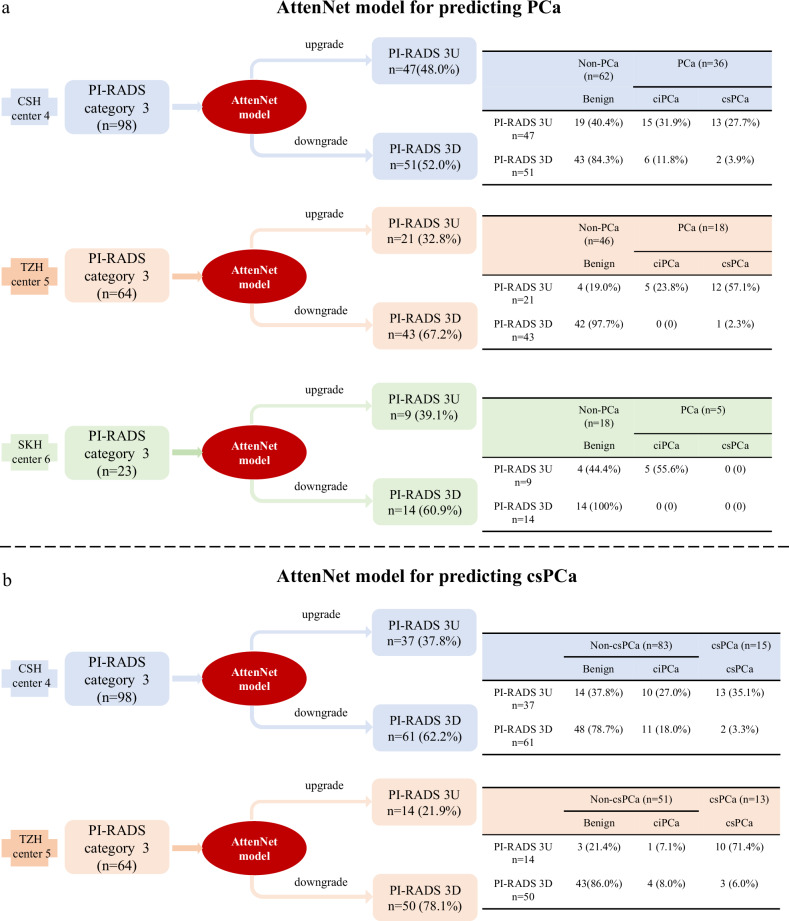
Clinical practice of AttenNet models for predicting PCa and csPCa in PI-RADS category 3 patients. **a** The downgrading and upgrading results of PI-RADS category 3 patients using the AttenNet model for predicting PCa in three external testing cohorts of center 4, center 5, and center 6. **b** The downgrading and upgrading results of PI-RADS category 3 patients using the AttenNet model for predicting csPCa in two external testing cohorts of center 4 and center 5. PI-RADS, prostate imaging-reporting and data system version; PI-RADS 3U, PI-RADS category 3 upgrade; PI-RADS 3D, PI-RADS category 3 downgrade; center4, CSH, Changshu No.1 People’s Hospital; center5, TZH, People’s Hospital of Taizhou; center6, SKH, Suzhou Kowloon Hospital; PCa, prostate cancer; ciPCa, clinically insignificant prostate cancer; csPCa, clinically significant prostate cancer

Figure 5 shows more details of the prediction results of AttenNet models from the clinical practice perspective. As shown in Fig. 5a, in each external testing cohort, the PI-RADS 3 patients were upgraded to PI-RADS 3U and downgraded to PI-RADS 3D according to the prediction results of the PCa by AttenNet model (i.e., PCa or non-PCa), respectively. The detailed results in each external testing cohort are described in Supplementary Section 6. As shown by Fig. 5a, 69.4% (Fig. 5a, center 4: 43/62) to 91.3% (Fig. 5a, center 5: 42/46) of benign (i.e., non-PCa) patients were identified by our AttenNet model from PI-RADS 3 patients of the external testing cohorts. In other words, these benign patients would have been spared from various clinical therapies and anxieties if the AttenNet model had been used to diagnose PCa.

As shown in Fig. 5b, in each external testing cohort, PI-RADS 3 patients were upgraded and downgraded to PI-RADS 3U and PI-RADS 3D according to the prediction results of csPCa by AttenNet model (i.e., csPCa and non-csPCa), respectively. The detailed results in each external testing cohort were described in Supplementary Section 7. As shown in Fig. 5b, 71.1% (Fig. 4b, center 4: [48 + 11]/83) to 92.2% (Fig. 5b, center 4: [43 + 4]/51) of non-csPCa were identified by our AttenNet model from the equivocal PI-RADS 3 patients of the external testing cohorts, all of whom had undergone biopsies because these non-csPCa patients could not be identified using PI-RADS assessment. Those results suggest that our AttenNet model has the potential to reduce unnecessary biopsies for non-csPCa patients.

### The results of subgroup analysis with AttenNet models for different levels of tumor size and PSA

**Fig. 6:**
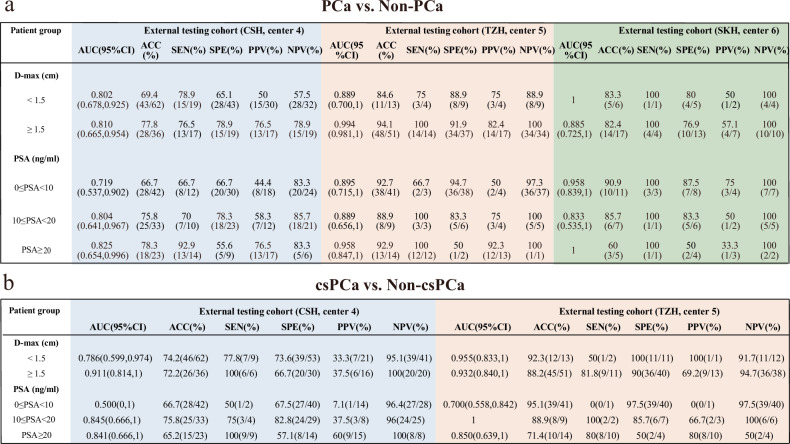
The results of subgroup analysis with AttenNet models for different levels of tumor size and PSA**. a** The performance of the AttenNet model for predicting PCa in each subgroup. **b** The performance of the AttenNet model for predicting csPCa in each subgroup*.* Center 4, CSH, Changshu No.1 People’s Hospital; center 5, TZH, People’s Hospital of Taizhou; center 6, SKH, Suzhou Kowloon Hospital; PCa, prostate cancer; csPCa, clinically significant prostate cancer; PSA, prostate-specific antigen; ROC, receiver operating characteristics; AUC, area under ROC curve; ACC, accuracy; SEN, sensitivity; SPE, specificity; D-max, diameter in greatest dimension

Additionally, we assessed the performance of the AttenNet models for predicting PCa and csPCa in subgroups of PI-RADS 3 patients according to different levels of tumor size and PSA. As revealed by the subgroup analysis (Fig. 6), the AttenNet models for predicting PCa and csPCa achieved satisfactory performance in different levels of D-max and PSA (except for the subgroup of 0 ≤ PSA < 10 ng/mL for predicting csPCa in the center 4) (Supplementary Section 8).

## Discussion

The present study developed DL models with attention modules (e.g., AttenNet), which achieved excellent performance for detecting both PCa and csPCa in external testing cohorts. The performance of our AttenNet model for detecting csPCa in PI-RADS 3 showed similar ACC to previous models assessing PI-RADS 1–5 patients [26, 27]. When our proposed model was used in clinical practice, 71.1–92.2% of non-csPCa patients were identified by our deep learning model in two testing cohorts, who can safely spare from invasive biopsy or radical prostatectomy (RP) procedure. These findings suggested that the proposed AttenNet models may be a promising tool to aid the precise risk stratification of PI-RADS 3 patients. To the best of our knowledge, this study first applied DL methodology to predict PCa and csPCa in PI-RADS 3 lesions. In contrast to some previous radiomics studies based on the manual segmentation and annotation of prostatic lesions for PI-RADS 3 patients [10–14], the present study can automatically mine the deep features of the lesions and their periphery and therefore is more conveniently applied to clinical practice.

Some recent studies have used deep learning methods to explore the discrimination between csPCa and non-csPCa, and achieved excellent diagnosis performance [27–29]. However, these studies developed the categorization models based on the prostate lesions with PI-RADS 1–5 rather than PI-RADS 3. As an extension of these studies, the present study not only constructed deep learning models with additional soft and channel attention modules, but also applied a transferring learning frame to achieve categorizations from csPCa and PCa. The soft attention module integrates the location information of lesions as clinical prior into the AttenNet model. According to reported guidelines of PI-RADS 2.1 [6], T2WI and DWI scores play a dominant role in the clinical assessment of prostate lesions when the lesions were in TZ and PZ, respectively. Additionally, the use of contrast contributes additional costs and time, as well as the potential risk of gadolinium-based contrast administration complications. It has been proposed that these clinical risks and burdens could be reduced by eliminating the dynamic contrast enhanced sequence altogether. Indeed, in this study, we only included the biparametric MRI data in the module training. The soft attention module increases the weight of corresponding biparametric MRI images in the output of the AttenNet model according to the location of the lesions. This modulatory processing is consistent with the evaluation criterion for the weight of sequences by PI-RADS assessed by radiologists in clinical practice and makes full use of the various information provided by multi-modal MRI images. The channel attention module can highlight important information throughout the pre-training and training processes by filtering out features that do not contribute to the classification, thereby helping to achieve accurate output scores. As demonstrated by the ablation experiments results, these attention modules guided the AttenNet model to focus on the critical features, thereby achieving excellent diagnostic performance for predicting PCa and csPCa.

In clinical practice, whether PI-RADS 3 patients require a biopsy is still debatable. In fact, as a routine practice in the clinical diagnosis of PCa [6, 7], PI-RADS 3 patients in the present study were suspected of high risks and therefore underwent biopsies to avoid missed diagnoses. However, such indiscriminate suspicions led to the over-biopsies of 84.7% (83/98) and 79.7% (51/64) in the external testing cohort of centers 4 and 5 respectively. In contrast, 71.1% and 92.2% of non-csPCa were identified by our proposed AttenNet model based on MRI images in center 4 and center 5 cohorts, respectively. In other words, the AttenNet model may avoid unnecessary biopsies for some patients. Thus, the AttenNet model has great potential to improve the SPE of the diagnosis of csPCa based on MRI images and therefore, to decrease the risk-benefit ratio of biopsy for PI-RADS 3 patients. The AttenNet model can be regarded as a triage test to decide which patients should undergo biopsy and which patients could safely avoid immediate painful biopsy.

For PI-RADS assessment based on T2WI and DWI, one of the important differences between PI-RADS 4 and 5 is the D-max (< 1.5 cm and ≥ 1.5 cm,) [6]. In the present study, the AttenNet models for predicting csPCa and PCa achieved satisfactory AUCs in both D-max < 1.5 cm and D-max ≥ 1.5 cm subgroups in all external testing cohorts. The PSA level of > 20 ng/mL had a high probability of PCa, whereas that within 0–20 ng/mL is associated with PCa incidence of less than 25% [30]. In addition to these two levels, even in moderate levels of PSA (i.e., 10–20 ng/mL), the AttenNet model achieved satisfactory discriminating performance in all external testing cohorts. This excellent performance of the AttenNet models can help increase radiologists’ diagnostic confidence.

There are several limitations in the present study. First, the sample sizes were not the same at all centers. Second, even though this is a multicenter retrospective study, a prospective multicenter study with more image data is needed in future research. Third, the data were from different MRI scanners. In the future, we will do more research on this issue. Fourth, not all patients underwent RP treatment for different clinical reasons; for some patients, biopsy pathology was used as a standard reference. In fact, some studies have reported that biopsy is a reliable way to detect PCa. Finally, although patients identified by this model were considered safe for the time being, follow-ups like repeat MRI or PSA should need to be taken seriously.

In conclusion, The AttenNet models with double channel attention modules achieved excellent performance in predicting both PCa and csPCa in PI-RADS 3 patients. Therefore, this model can greatly reduce unnecessary biopsies and thereby improve the risk-benefit ratio of biopsy for PI-RADS 3 patients.

## Supplementary information

ELECTRONIC SUPPLEMENTARY MATERIAL

## Data Availability

The imaging studies and clinical data used for algorithm development are not publicly available because they contain private patient health information. Interested users may request access to these data, where institutional approvals, along with signed data use agreements and/or material transfer agreements may be needed/negotiated. Derived result data supporting the findings of this study are available upon reasonable request.

## Abbreviations

ACC: Accuracy
ADC: Apparent diffusion coefficient
AttentNet: ResNet combined with transfer, channel attention and soft attention modules
AUC: Area under the ROC curve
ciPCa: Clinically insignificant prostate cancer
csPCa: Clinically significant prostate cancer
DCA: Decision curve analysis
D-max: Diameter in greatest dimension
IQR: Interquartile range
ISUP: International Society of Urological Pathology
mpMRI: Multiparametric magnetic resonance imaging
PCa: Prostate cancer
PI-RADS: Prostate imaging reporting and data system
PSA: Prostate specific antigen
PZ: Peripheral zone
ResNet-T: ResNet combined with transfer module
ResNet-TC: ResNet combined with transfer and channel attention module
ROC: Receiver operating characteristic
RP: Radical prostatectomy
SEN: Sensitivity
SPE: Specificity
TRUS: Transrectal ultrasound
TZ: Transitional zone
