# Supplementary material for: 3D-AttenNet model can predict clinically significant prostate cancer in PI-RADS category 3 patients: a retrospective multicenter study
Source: Insights Imaging. 2025 Jan 29;16:25. doi: 10.1186/s13244-024-01896-1 (PMC11780012; doi:10.1186/s13244-024-01896-1)
Supplement: Supplementary file 1 — ELECTRONIC SUPPLEMENTARY MATERIAL [file 13244_2024_1896_MOESM1_ESM.pdf]

3D-AttenNet Model Can Predict Clinically Significant Prostate Cancer in PI-RADS Category 3 Patients: A Retrospective Multicenter Study

ELECTRONIC SUPPLEMENTARY MATERIAL

Table S1. The baseline characteristics of pretraining cohorts, training cohorts and external testing cohorts

Variables	Pretraining and Training cohorts (center 1-3, n = 1382)			External testing cohorts (center 4-6, n = 624)		
	SUH1st (Center1, n=705)	SUH2nd (Center2, n=550)	ZJGH (Center3, n=127)	CSH (Center 4, n=280)	TZH (Center 5, n=248)	SKH (Center6, n=96)
No. of subjects	705	550	127	280	248	96
Age (y), mean	69±8.2	69.5±8.4	75.3±4.7	70.0±7.0	72.7±7.8	70.0±8.1
PSA level, median (IQR)	13.81 (8.1-31.0)	10.16 (6.5-15.6)	17.8 (11.0-59.2)	17.0 (15.2)	15.5 (6.3-71.9)	13.4 (6.7-55.1)
0-10 ng/ml, n (%)	247 (35.0%)	268 (48.7%)	26 (20.5%)	109 (38.9%)	99 (39.9%)	38 (39.6%)
10-20 ng/ml, n (%)	204 (28.9%)	189 (34.4%)	41 (32.3%)	88 (31.4%)	30 (12.1%)	14 (14.6%)
＞20 ng/ml, n (%)	254 (36.0%)	93 (16.9%)	60 (47.2%)	83 (29.6%)	119 (48%)	44 (45.8%)
D-max (mm), median (IQR)	23.3 (17.8-33.7)	18.9 (14.4-24.6)	22.5 (15.1-37.7)	10.1 (10.2)	40.9 (28.3-52.0)	11.0 (8.0-16.0)
Prostate Zone, n	705	550	127	280	248	96
PZ, n (%)	345 (48.9%)	146 (26.5%)	45 (35.4%)	69 (24.6%)	37 (14.9%)	34 (35.4%)
TZ, n (%)	238 (33.8%)	311 (56.5%)	58 (45.7%)	175 (62.5%)	122 (49.2%)	62 (64.6%)
PZ and TZ, n (%)	122 (17.3%)	93 (16.9%)	24 (18.9%)	36 (12.9%)	89 (35.9%)	0 (0.0%)
PI-RADS of index lesion per patient, n (%)	705	550	127	280	248	96
PI-RADS 1-2	142(20.1%)	260 (47.3%)	31 (24.4%)	98 (35.0%)	68 (27.4%)	22 (22.9%)
**PI-RADS 3**	**82(11.6%)**	**129 (23.5%)**	**27 (21.3%)**	**98 (35.0%)**	**64 (25.8%)**	**23 (24.0%)**
PI-RADS 4	125(17.7%)	99 (18.0%)	14 (11.0%)	20 (7.1%)	25 (10.1%)	25 (26.0%)
PI-RADS 5	356(50.5%)	62 (11.3%)	55 (43.3%)	64 (22.9%)	91 (36.7%)	26 (27.1%)
Biopsy ISUP grade, n (%)	685	550	127	275	248	80
GG0 (Benign)	179 (26.1%)	374 (68.0%)	47 (37.0%)	165 (60.0%)	105 (42.3%)	43 (53.8%)
GG1	58 (8.5%)	66 (12.0%)	17 (13.4%)	7 (2.5%)	8 (3.2%)	9 (11.3%)
GG2	124 (18.1%)	27 (4.9%)	17 (13.4%)	40 (14.5%)	16 (6.5%)	12 (15.0%)
GG3	127 (18.5%)	26 (4.7%)	10 (7.9%)	17 (6.2%)	30 (12.1%)	2 (2.5%)
GG4	91 (13.3%)	22 (4.0%)	16 (12.6%)	34 (12.4%)	46 (18.5%)	9 (11.3%)
GG5	106 (15.5%)	35 (6.4%)	20 (15.7%)	12 (4.4%)	43 (17.3%)	5 (6.3%)
Surgical ISUP grade, n (%)	425	112	20	5	26	28
GG1	31 (7.3%)	45 (40.2%)	5 (25.0%)	0 (0.0%)	0 (0.0%)	3 (10.7%)
GG2	103 (24.2%)	22 (19.6%)	5 (25.0%)	2 (40.0%)	6 (23.1%)	10 (35.7%)
GG3	132 (31.1%)	19 (17.0%)	2 (10.0%)	2 (40.0%)	3 (11.5%)	2 (7.1%)
GG4	48 (11.3%)	12 (10.7%)	2 (10.0%)	1 (20.0%)	6 (23.1%)	7 (25.0%)
GG5	111 (26.1%)	14 (12.5%)	6 (30.0%)	0 (0.0%)	11 (42.3%)	6 (21.4%)
Label, n (%)	705	550	127	280	248	96

non-PCa	179 (25.4%)	374 (68.0%)	47 (37.0%)	165 (58.9%)	109 (44.0%)	48 (50.0%)
PCa	526 (74.6%)	176 (32.0%)	80 (63.0%)	115 (41.1%)	139 (56.0%)	48 (50.0%)
Non-csPCa	328 (46.5%)	440 (80.0%)	77 (61.4%)	214 (76.4%)	128 (51.6%)	73 (76.0%)
csPCa	377 (53.5%)	110 (20.0%)	49 (38.6%)	66 (23.6%)	120 (48.4%)	23 (24.0%)
ECE, n (%)	425	112	20	5	26	28
Present	116 (27.3%)	20 (17.9%)	5 (25.0%)	1 (20.0%)	12(46.2%)	7 (25.0%)
Absent	309 (72.7%)	92 (82.1%)	15 (75.0%)	4 (80.0%)	14(53.8%)	21 (75.0%)
SVI, n (%)	425	112	20	5	26	28
Present	54 (12.7%)	10 (8.9%)	2 (10.0%)	0 (0.0%)	5 (19.2%)	4 (14.3%)
Absent	371 (87.3%)	102 (91.1%)	18 (90.0%)	5 (100.0%)	21 (80.8%)	24 (85.7%)
LNI, n (%)	146	30	0	2	26	23
Present	13 (8.9%)	3 (10.0%)	0 (0.0%)	0 (0.0%)	0 (0.0%)	5 (21.7%)
Absent	133 (91.1%)	27 (90.0%)	0 (0.0%)	2 (100.0%)	26 (100.0%)	18 (78.3%)

Note. Unless indicated otherwise, data are numbers of patients with percentage in parentheses. P-value was evaluated by two-tailed t-test with unequal variance. Gleason grade (GG) is according to the 2014 International Society of Urological Pathology (ISUP) standards.
ciPCa = clinically insignificant prostate cancer; csPCa= clinically significant prostate cancer; PZ = peripheral zone; TZ = transition zone; CZ = center zone; AFMS = anterior fibromuscular stroma; ECE = extracapsular extension; SVI = seminal vesicle infiltration; LNI = lymph node invasion; D-max= diameter in greatest dimension

Section 1. Prostatic MRI Scanning

Scan protocols included T1 weight imaging (T1WI) (transverse), T2 weight imaging (T2WI) (transverse, coronal and sagittal), diffusion-weighted imaging (DWI) (transverse), and dynamic contrast-enhanced (DCE). The apparent diffusion coefficient (ADC) value was calculated from DWI using a monoexponential model. After a routine MRI examination, a T1WI, 3D gradient recalled echo protocol was performed to acquire DCE imaging data. The parameters of each scanning sequence of each institute were summarized in **Table S2**.

Table S2. Parameters of MRI scanning from six institutions

Center	Sequences	Vendor	MRI strength	B value(sec/mm^2)	Slice thickness (mm)	Spacing between slices (mm)	Echo time(s)	Repetition time(s)
SUH1st	T2WI, DWI, ADC	Siemens Skyra (Erlangen, Germany)	3.0 T	50/70/1500/2000	3	3/3.45	60/104	6540/7590
SUH 2nd	T2WI, DWI, ADC	Philips Ingenia (Best, the Netherlands)	3.0 T	10/20/50/100/200/1000/2000	1.5/2/3/3.4/3.5/3.7/ 3.8/3.9/4/4.1/4.2/4.3/5	1.65/3/3.2/3.3/3.4/3.5/3.7/ 3.8/3.9/2.2/4/4.1/4.2/4.3/5	77/78/100	4542/4828/4898/ 4733/4972/ 6000
ZJGH	T2WI, DWI, ADC	Philips achieva (Best, the Netherlands)	3.0 T	0/400/800/1500	3	3.3	62/75	20004342//6118/ 6173/6498/6489
SKH	T2WI, DWI, ADC	Siemens Skyra (Erlangen, Germany)	3.0 T	400/800/1200	3.5/4	3.5/4	72/97	3900/4342/6118/ 6173/6489/6498/ 7500
TZH	T2WI, DWI, ADC	Siemens Skyra and Vero (Erlangen, Germany)	3.0 T	0/50/800/1000/1500	3.5/4/5/5.5	3.5/4/4.8/6/6.6	62/64/74/97/104	4480/5000/5100/ 7500/8600
CSH	T2WI, DWI, ADC	Philips Achieva TX (Best, the Netherlands)	3.0 T	0, 1000, 2000	3	3	76/80	2750/3000

Notes: T2WI, T2-weighted imaging; DWI, diffusion-weighted imaging; ADC, Apparent diffusion coefficient; SUH1st, the first affiliated hospital of Soochow University; SUH2nd, the second affiliated hospital of Soochow University; ZJGH, People's Hospital of Zhangjiagang; CSH, Changshu NO.1 People's Hospital; TZH, People's Hospital of Taizhou; SKH, Suzhou Kowloon Hospital

Section 2. PI-RADS assessment

If a patient had multiple lesions, the index lesion referred to the lesion with the highest Gleason score or the largest size (if the lesions had the same Gleason score). Only the index lesions were used in the present study. First, all included patients were divided into five groups, and their MRI images were assessed by five board-certified radiologists (with 3-5 years of experience in prostatic MRI assessment) according to the criteria of PI-RADS v2.1 [1]. Then, the results of the PI-RADS assessment were divided into two groups and checked by two expert-level radiologists (with 18- and 22- years of experience in prostatic MRI assessment), if there are inconsistencies in the scores, the radiologists discussed until an agreement is reached. During the PI-RADS assessment, all readers were blinded to the pathological information but aware of clinical information such as age, prostate-specific antigen (PSA) level, digital

rectal examination, and other risk factors such as family history and routine habits. In addition, the discrepancies in PI-RADS scores of the index lesions among the radiologists were discussed to reach an agreement. Manual segmentations of all patients in both training and testing cohorts were segmented on T2W images by the above 5 readers. Because the tumor boundary in MRI was too ambiguous to be clearly detected, the VOIs were segmented twice by the five radiologists. The authorized VOIs of the targeted lesions were deemed as the regional identification overlapping in two instances. The VOIs delineated on T2W images were used as a mask to extract corresponding lesions on DWI and ADC images.

Section 3. Histopathologic review

The systemic ultrasound guided biopsy used the transperineal/transrectal route to obtain 12 cores, and the targeted MRI-guided biopsy or cognitive fusion biopsy obtained additional 2 or more cores according to the number of suspected tumors. All the biopsy procedures were performed by urologists, and all the urologists revised the available histopathological slides according to the 2014 International Society of Urological Pathology (ISUP) guidelines [1, 2]. The patients with positive biopsy findings during the present study were recommended for radical prostatectomy (RP). Thus, for patients undergoing both biopsy and RP or only RP, RP pathology was used as the final ground truth. For patients without RP, biopsy interpretation was used as the ground truth. All histopathology findings were graded according to their respective

Gleason scores (GS), namely ISUP grade 0 (GS < 3 + 3), ISUP grade 1 (GS = 3 + 3), ISUP grade 2 (GS = 3 + 4), ISUP grade 3 (GS = 4 + 3), ISUP grade 4 (GS = 8), and ISUP grade 5 (GS > 8). In terms of histopathology, the patients with ISUP <1 and ISUP ≥ 1 were defined as benign (i.e., non-PCa group) and malignant (i.e., PCa group), respectively. Patients with ISUP <3 and ISUP ≥ 3 were defined as the non-csPCa group and csPCa group, respectively. Additionally, Patients with ISUP = 1 and 2 were defined as clinically insignificant prostate cancer (ciPCa) according to the reference [3, 4].

Section 4. MRI data preprocessing

Data de-identification and registration

All multiparameter MRI images were first converted from Digital Imaging and Communications in Medicine (DICOM) to Neuroimaging Informatics Technology Initiative (NIFTI) format to remove patients' private information.

After preprocessing, for each patient, a 3D ROI, including the index lesion, was produced with a resolution of 112×112×16, which met the dimension of the input of the DL model. For each patient, the DWI images and ADC maps derived from DWI with different gradient field parameters were spatially aligned to the T2WI images by a rigid 3D registration method using the SimpleElastix toolbox (http://simpleelastix.github.io/).

Data harmonization

Additionally, even in the same center, the size of index lesions also presented great inter-patient variation. The aim of data harmonization is to resample the cuboid 3D regions of interest (ROI) containing the entire index lesion into the common spatial resolution for all patients and thereby meet the dimension of the input of the deep learning model.

The data harmonization included three steps: (1) all MRI images were resampled to a common voxel size of 0.46×0.46×3, which was the median value of voxel spacing in the training cohort; (2) for each index lesion, in the slice where the section of the lesion was the largest extent size, a 2D square ROI was produced to comprise the lesion with an additional 5-voxel margin. Then, this 2D ROI was reproduced in the slices containing the lesion with an additional 5-slice margin extending to the top and bottom. Finally, these 2D ROI consisted of a 3D ROI. (3) For all patients, the 3D ROIs were resampled into a common resolution of 112×112×16. (3) For each 3D ROI, the intensity of each voxel was converted to z-score, namely $z\text{-}score = \frac{x_i - \bar{x}}{\sigma}$, where x_i is the original intensity value of the *i*th voxel, and $\bar{x}$ and σ are the mean and standard deviation across all voxels of corresponding 3D ROI, respectively.

Data augmentation

In order to prevent models from over-fitting and further improve models' generalization, the data of the pretraining and training cohorts (except for their respective tuning datasets) was augmented by the translations in random directions and rotations at random angles. The augmented pretraining and training cohorts, and not-augmented tuning datasets were employed to develop deep learning models. The remaining not-augmented testing cohorts were used to test models' performance.

Section 5 Model architecture

AttenNet consisted of three parallel and independent branches with the 3D ROIs of T2WI, DWI images and ADC maps as inputs, respectively, which used the state-of-art ResNet3D as the basic network due to its ability to mine features of deep layers and generate accurate predicting values using shortcut connections [5].

For the branch with the input of T2WI images, the 3D ROIs of T2WI were first fed into a module of ResNet3D with channel attention (**Figure 2a**). Then, the last layer features extracted from this module were further fed into a channel attention module (**Figure 2a**). Finally, a fully connected layer mapped the refined features extracted from the channel attention module into a one-dimension output score (**Figure 2a**). In this branch, the module of ResNet3D with channel attention included a convolutional layer, a batch normalization

layer, an activation function of rectified linear unit (Relu) and four blocks (**Figure 2b**). Each of these four blocks included two convolutional layers, two batch normalization layers, two-channel attention modules, and an activation function of Relu (**Figure 2c**). The architectures of the other two branches were the same as that of the branch with the input of T2WI, except that the T2WI images were respectively replaced by DWI and ADC images. In this deep learning model, in contrast to the other modules, the channel attention modules can learn the importance of each feature channel during the training process, thereby enhancing the feature channels associated with the classification and, simultaneously, suppressing the feature channels irrelevant to the classification. Thus, the involvement of channel attention modules can make the model adaptively guide feature extractions and highlight important information for predicting PCa and csPCa.

A fusion network integrated these three branches with the involvement of the soft attention module (**Figure 2a**). The present study used the soft attention module to provide the location information of prostate lesions for the AttenNet model. Specifically, the location information of prostate lesions was used as a clinical prior and encoded into the Embedding (i.e., three vectors of α_{PZ}, α_{TZ}, α_{PZ+TZ}), which corresponded to different lesion locations (i.e., only PZ, only TZ, and both PZ and TZ), respectively (**Figure 2a**). Each vector of the Embedding included three elements, which indicated the weights of the branch networks with inputs of T2WI, DWI, and ADC images during the integration of the branch

networks in the fusion network. As shown in **Figure 2a**, the Embedding vectors were initialized at the beginning of pre-training and training processes according to the following criteria. If the lesion was only in TZ, the $\boldsymbol{\alpha}_{TZ}$ was initialized as [1, 0.5, 0.5], and if the lesion was only in PZ, the $\boldsymbol{\alpha}_{PZ}$ was initialized as [0.5, 1, 1]. Further, if the lesion was in both PZ and TZ, the $\boldsymbol{\alpha}_{PZ+TZ}$ was initialized as [1, 1, 1]. Then the values of the elements of Embedding vectors were automatically and iteratively changed during the pretraining and training processes.

As shown in **Figure 2**, the output scores of the branch networks with inputs of T2WI, DWI, and ADC images were defined as the elements of y_b = (y_{T2WI}, y_{DWI}, y_{ADC}), respectively. Then, the output score y' was calculated by the inner production of y_b and one of Embedding vectors selected according to the location information of the lesion. Finally, the risk probability y was obtained by scaling y' to a range of 0 to 1 through the softmax layer. For example, if the lesion was in PZ, the risk probability y was calculated as follows:

$$y' = (\alpha_{PZ,\,0} \times y_{T2WI} + \alpha_{PZ,\,1} \times y_{DWI} + \alpha_{PZ,\,2} \times y_{ADC})$$

$$y = \text{Softmax}\ (y') \in [0,1]$$

where $\alpha_{PZ,\,0}$, $\alpha_{PZ,\,1}$ and $\alpha_{PZ,\,2}$ are the elements of Embedding vector of $\boldsymbol{\alpha}_{PZ}$. On each of pretraining and training steps, cross-entropy loss function (i.e., L) was as follows:

$$L = \frac{1}{N}\sum_{i} L_i = \frac{1}{N}\sum_{i} -[Y_i \cdot \log(y_i) + (1 - y_i) \cdot \log(1 - y_i)]$$

where, *i* represents the patient ID and *N* refers to the number of samples in the pretraining or training cohort. *Y* was the label (i.e., ground truth).

Section 6. Clinical Practice of the AttenNet Models for Predicting PCa in PI-RADS Category 3 Patients

Furthermore, in 21.8% (52/238) patients, lesions were located only in PZ, in 67.2% (160/238) cases in TZ, and in both TZ and PZ in 10.9% (26/238) of patients.

In the external testing cohorts (center 4~6), 31.9% (59/185) and 68.1% (126/185) patients were diagnosed as PCa and benign (non-PCa), respectively, and 15.1% (28/185) and 84.9% (157/185) as csPCa and non-csPCa, respectively. Also, 18.4% (34/185) patients had lesions in PZ, 74.1% (137/185) patients in TZ, and 7.6% (14/185) patients in both PZ and TZ.

As indicated by **Table 1**, the difference in the distribution of surgical ISUP grade between training and testing cohorts was significant ($P < 0.001$). There was no difference in the age, PSA level or diameter in the greatest dimension (D-max) between the training and testing cohorts (all $P > 0.05$). For the patients

with RP, there was no difference in the presence of extracapsular extension (ECE), seminal vesicle infiltration (SVI) or lymph node invasion (LNI) between the training and testing cohorts (Ps > 0.05).

In center 4, 48.0% (47/98) of patients were upgraded to PI-RADS 3U, of which 59.6% ([15+13]/47) and 40.4% (19/47) were histopathologically confirmed with PCa and non-PCa by histopathological exams, respectively. In contrast, 52.0% (51/98) of patients were downgraded to PI-RADS 3D, of which 15.7% ([6+2]/51) and 84.3% (43/51) were histopathologically confirmed with PCa and non-PCa, respectively. As a result, in center 4, when the AttenNet model was performed on the PI-RADS 3 patients, 69.4% (43/62) patients were recognized as non-PCa and 77.8% ([15+13]/36) patients were recognized as PCa.

In center 5, 32.8% (21/64) patients were upgraded to PI-RADS 3U, of which 81.0% ([5+12]/21) and 19.0% (4/21) were histopathologically confirmed with PCa and non-PCa, respectively. In contrast, 67.2% (43/64) patients were downgraded to PI-RADS 3D, of which 2.3% (1/43) and 97.7% (42/43) were histopathologically confirmed with PCa and non-PCa, respectively. As a result, in center 5, when the AttenNet model was performed on the PI-RADS 3 patients, it achieved a specificity of 91.3% (42/46) and a sensitivity of 94.4% ([5+12]/18) for predicting PCa.

In center 6, 39.1% (9/23) patients were upgraded to PI-RADS 3U, of which 55.6% (5/9) and 44.4% (4/9) were histopathological confirmed with PCa and non-PCa, respectively. In contrast, 60.9% (14/23) patients were downgraded to PI-RADS 3D, of which all (14/14) patients had non-PCa. As a result, when the AttenNet model was performed on the PI-RADS 3 patients, it achieved a specificity of 77.8% (14/18) and a sensitivity of 100% (5/5) for predicting PCa.

Section 7. Clinical Practice of the AttenNet Models for Predicting csPCa in PI-RADS Category 3 Patients

As shown in **Figure 4b**, in center 4, 37.8% (37/98) patients were ungraded to PI-RADS 3U, of which 35.1% (13/37) and 64.9% ([14+10]/37) patients were histopathologically confirmed with csPCa and non-csPCa, respectively. In contrast, 62.2% (61/98) patients were downgraded to PI-RADS 3D, of which 3.3% (2/61) and 96.7% ([48+11]/61) were histopathologically confirmed with csPCa and non-csPCa, respectively. As a result, when the AttenNet model was performed on PI-RADS 3 patients, it achieved a specificity of 71.1% ([48+11]/83) and a sensitivity of 86.7% (13/15) for predicting csPCa.

In center 5, 21.9% (14/64) patients were upgraded to PI-RADS 3U, of which 71.4% (10/14) and 28.6% ([3+1]/14) patients were histopathological confirmed with csPCa and non-csPCa, respectively. In contrast, 78.1% (50/64) patients were downgraded to PI-RADS 3D, of which 6% (3/50) and 94% ([43+4]/50) patients were histopathological confirmed with csPCa and non-csPCa,

respectively. As a result, when the AttenNet model was performed on the PI-RADS 3 patients, it achieved a specificity of 92.2% ([43+4]/51) and a sensitivity of 76.9% (10/13) for predicting csPCa.

Section 8. The Results of Subgroup Analysis with AttenNet Models for Different Levels of Tumor Size and PSA

The AttenNet models for predicting PCa and csPCa achieved satisfactory performance in different levels of D-max and PSA (except for the subgroup of 0 ≤ PSA < 10 ng/ml for predicting csPCa in the center 4). As shown in **Figure 5a**, in the external testing cohorts of center 4, center 5 and center6, the AttenNet model for predicting PCa achieved AUCs of 0.802 (95%CI, [0.678-0.925]), 0.889 (95%CI, [0.700-1]), and 1 in a subgroup of D-max < 1.5 cm; 0.810 (95%CI, [0.665-954]), 0.994 (95%CI, [0.981-1]) and 0.885 (95%CI, [0.725-1]) in a subgroup of D-max ≥1.5 cm, respectively.

In terms of PSA, in the external testing cohorts of center 4, center 5 and center6, the AttenNet model for predicting PCa achieved AUCs of 0.719 (95%CI, [0.537-0.902]), 0.895 (95%CI, [0.715-1]), and 0.958 (95%CI, [0.839-1]) in the subgroup of 0≤ PSA< 10 ng/ml; 0.804 (95%CI, [0.641-0.967]), 0.889 (95%CI, [0.656-1]), and 0.833 (95%CI, [0.535-1]) in the subgroup of 10 ≤ PSA <20 ng/ml; 0.825 (95%CI, [0.654-0.996]), 0.958 (95%CI, [0.847-1]), and 1 in the subgroup of PSA ≥ 20 ng/ml, respectively (**Figure 5a**).

As shown in **Figure 5b**, in external testing cohorts of center 4 and center 5, the AttenNet model for predicting csPCa achieved AUCs of 0.786 (95%CI, [0.599-0.974]), and 0.955 (95%CI, [0.833-1]) in a subgroup of D-max < 1.5cm, 0.911(95%CI, [0.814-1]) and 0.932(95%CI, [0.840-1]), respectively. In terms of PSA, in the external testing cohorts of center 4 and center 5, the AttenNet model for predicting csPCa achieved AUCs of 0.500 (95%CI, [0-1]) and 0.700 (95%CI, [0.558-0.842]) in a subgroup of 0 ≤ PSA < 10 ng/ml, 0.845(95%CI, [0.666-1]) and 1 in a subgroup of 10 ≤ PSA < 20 ng/ml, and 0.841(95%CI, [0.666-1]) and 0.850 (95%CI, [0.639-1]) in the subgroup of PSA ≥ 20 ng/ml, respectively.

References

1. Turkbey, B., Rosenkrantz, A. B., Haider, M. A., Padhani, A. R., Villeirs, G., Macura, K. J. *et al.* Prostate Imaging Reporting and Data System Version 2.1: 2019 Update of Prostate Imaging Reporting and Data System Version 2. *European Urology* **76**, 340-351 (2019).
2. Epstein, J. I., Egevad, L., Amin, M. B., Delahunt, B., Srigley, J. R. & Humphrey, P. A. J. T. A. J. o. S. P. The 2014 international society of urological pathology (ISUP) consensus conference on gleason grading of prostatic carcinoma definition of grading patterns and proposal for a new grading system. **40**, 244-252 (2015).
3. Ahmed, H. U., El-Shater Bosaily, A., Brown, L. C., Gabe, R., Kaplan, R., Parmar, M. K. *et al.* Diagnostic accuracy of multi-parametric MRI and TRUS biopsy in prostate cancer (PROMIS): a paired validating confirmatory study. *The Lancet* **389**, 815-822 (2017).
4. Ahdoot, M., Wilbur, A. R., Reese, S. E., Lebastchi, A. H., Mehralivand, S., Gomella, P. T. *et al.* MRI-Targeted, Systematic, and Combined Biopsy for Prostate Cancer Diagnosis. *N Engl J Med* **382**, 917-928 (2020).
5. Niethammer, M., Kwitt, R. & Vialard, F. X. Metric Learning for Image Registration. *Proceedings. IEEE Computer Society Conference on Computer Vision and Pattern Recognition* **2019**, 8455-8464 (2019).
